# Optimal 1TEL–target protein linker character is target protein-dependent

**DOI:** 10.1107/S2059798326002494

**Published:** 2026-04-07

**Authors:** Maria J. Pedroza Romo, Alihikaua Keliiliki, Jacob C. Averett, Joseph F. Gonzalez, Ethan Noakes, Elijah W. Wilson, Conrad Smith, Blake Averett, Dalton Hansen, Riley Nickles, Miles Bradford, Sara Soleimani, Tobin Smith, Supeshala Nawarathnage, Prasadika Samarwickrama, Ariel Kelsch, Derick Bunn, Cameron Stewart, Wisdom Abiodun, Evan Tsubaki, Seth Brown, Tzanko I. Doukov, James D. Moody

**Affiliations:** a Department of Chemistry and Biochemistry Brigham Young University 701 East University Parkway Provo UT84602 USA; b Macromolecular Crystallography Group, Structural Molecular Biology Resource, Stanford Synchrotron Radiation Lightsource, Menlo Park, CA94025, USA; National Hellenic Research Foundation, Greece

**Keywords:** TELSAM, DARPins, ETV6, designed ankyrin-repeat proteins, protein binding, linkers, 1TEL

## Abstract

Fusing a variant of the sterile alpha motif domain of the human translocation ETS leukaemia protein (1TEL) to a protein of interest has been shown to significantly enhance its crystallization propensity. 1TEL is a pH-dependent, polymer-forming protein crystallization chaperone which, when covalently fused to a protein of interest, forms a stable, well ordered crystal lattice. However, despite its success, a challenge persists in that crystal quality and diffraction limits appear to be heavily dependent on the choice of linker between 1TEL and the protein of interest, with the identification of a functional linker currently relying on trial-and-error methods. Likewise, previous studies revealed that a ten-histidine tag at the 1TEL N-terminus can either facilitate or hinder the ordered crystallization of target proteins attached via flexible or semi-flexible linkers. To address these challenges, we designed multiple constructs with several types of linkers [rigid (helical fusion), semi-flexible (Pro-Ala and Pro-Ala-Ala) and flexible (Gly-Gly and Gly-Gly-Gly)] of varying lengths to fuse either a designed ankyrin-repeat protein (DARPin) or the thirty-eight-negative kinase-1 ubiquitin-associated (UBA) domain to the 1TEL C-terminus. Semi-flexible and flexible linker constructs were made with and without a ten-histidine tag. Our findings indicate that short semi-flexible and rigid linkers consistently yielded large crystals with a DARPin target protein, but that flexible linkers performed best with a UBA-domain target protein. Removing the ten-histidine tag uniformly enhanced crystallization rates, improved the crystal morphology and increased the crystallization propensity of the semi-flexible and flexible linker constructs. These results suggest that the ideal linker selection primarily depends on the properties of the target protein. Our data support our current recommendation to use a short flexible or semi-flexible linker between 1TEL and the target protein to facilitate protein crystallization and high-resolution structure determination.

## Abstract 2

In this study, we examine the effect of short to medium-length flexible, semi-flexible and rigid linkers on the crystallization of a DARPin or the TNK1 UBA domain fused to the 1TEL protein crystallization chaperone, demonstrating that while rigid linkers can impair crystallization and reduce diffraction quality, the ideal linker character remains target protein-dependent.

## 1. Introduction

Atomic-resolution macromolecular structures are vital for experimental structural characterization, which is essential for drug and target discovery, design, virtual screening, crystallographic drug fragment screening and elucidation of protein structure–function relationships in health and disease (Wei & McCammon, 2024 ▸; Ferreira *et al.*, 2021 ▸; Lionta *et al.*, 2014 ▸; Maveyraud & Mourey, 2020 ▸). X-ray crystallography remains the method of choice for obtaining high-resolution structures of macromolecules smaller than 50–100 kDa. Of particular use to drug developers are structures resolved to better than 2 Å resolution, because correct drug-docked configurations and binding-site waters can be readily resolved. Despite advancements in molecular cloning, protein production and purification, crystallization screening, synchrotron data collection and structure-determination software, obtaining diffraction-quality crystals remains the rate-limiting step in characterizing macromolecular structures through X-ray crystallography (Dale *et al.*, 2003 ▸; Terwilliger *et al.*, 2009 ▸; Cooper *et al.*, 2011 ▸).

To address this bottleneck, researchers have turned to innovative approaches, including the use of engineered fusion proteins to enhance crystallization efficiency (Derewenda, 2010 ▸). One of these is the human translocation ETS leukaemia (TEL, ETV6) sterile alpha motif (SAM) domain. A single amino-acid substitution of the TEL SAM domain (hereafter termed 1TEL) causes it to be soluble at pH ≳ 8 and to polymerize into well ordered helical polymers at pH ≲ 8. These polymers feature six 1TEL subunits per turn of the helix. Target proteins can be genetically fused to 1TEL, which promotes crystallization by ordering many copies of the target protein along ordered 1TEL polymers, which then associate to form a crystal. 1TEL fusion crystallization (TFC) offers many advantages in addressing classical protein crystallization challenges: TFC (i) decreases the entropic cost of crystal nucleation and growth, (ii) increases crystallization propensity (here defined as the fraction of crystallization conditions producing crystals), reproducibility and speed, (iii) enables crystallization at unusually low protein concentrations, (iv) yields crystals that reach the same diffraction limits as crystals from traditional methods, but with significantly fewer crystal contacts, and (v) does not perturb the target protein structure. TFC has been observed to achieve a success rate in forming crystals of up to 90% (Nauli *et al.*, 2007 ▸), promising a highly cost-effective approach for accelerating structural determination (Gajjar *et al.*, 2023 ▸; Nawarathnage *et al.*, 2022 ▸, 2023 ▸).

We previously created four protein constructs that fused this same DARPin (Seeger *et al.*, 2013 ▸) to 1TEL or 3TEL using either a long α-helical fusion or a Gly-Gly-Gly linker (Nawarathnage *et al.*, 2022 ▸). While 1TEL refers to the use of one TEL SAM domain per covalently fused target protein, 3TEL refers to three TEL SAM domains covalently fused in tandem, with one copy of the target protein fused to the C-terminus of the third SAM domain. All constructs gave crystals, but only the crystals of the 3TEL constructs were of diffraction quality. We questioned why the 1TEL constructs failed to yield large or diffracting crystals, but did not pursue this question further at the time. In a later study (Gajjar *et al.*, 2023 ▸) we discovered the importance of short, semi-flexible linkers in 1TEL–capillary morphogenesis gene 2 (CMG2) von Willebrand factor type A (vWA) constructs. Constructs with bulkier, less flexible linkers consistently yielded higher resolution diffraction data, leading us to hypothesize that optimizing the linkers in 1TEL–DARPin constructs could enable them to form larger, well ordered crystals. Additionally, another study (Kottur *et al.*, 2022 ▸) demonstrated that Pro-Ala and Pro-Ala-Ala linkers provided high-resolution structures of the SARS CoV2 nsp14 methyltransferase domain when using TFC. Here, we report our work optimizing the 1TEL–DARPin linkers, testing three types of linkers with varying lengths and residue identities: flexible linkers (GG and GGG), semi-flexible linkers (PA and PAA) and rigid α-helical fusion (0, 3 and 13 residues) linkers, as shown in Table 1 ▸. In this work, we also included a 1TEL–thirty-eight-negative kinase-1 (TNK1) ubiquitin-associated (UBA) domain system (1TEL–UBA) to identify more generalizable conclusions (Table 1 ▸).

## 2. Materials and methods

### 2.1. Cloning

A gene fragment that comprised the sequence of the designed ankyrin-repeat protein (DARPin) from PDB entry 4j7w (Seeger *et al.*, 2013 ▸) was placed downstream of residues 47–121 (the SAM domain) of the human translocation ETS leukaemia protein (TEL; UniProt P41212; PDB entry 2qar). This variant of the SAM domain bears a V112E substitution (that makes its polymerization pH-sensitive) and will be referred to as 1TEL hereafter. To form the flexible and semi-flexible linkers, Pro-Ala, Pro-Ala-Ala, Gly-Gly and Gly-Gly-Gly were inserted between the 1TEL and DARPin domains. To form the short-rigid linker, the first amino acid of the DARPin was deleted and the 1TEL and DARPin sequence fragments were directly fused. To form the medium-rigid linker, the amino acids Lys-Gln-Arg were inserted between the 1TEL and DARPin domains using *Geneious* (version 9.1.8; Dotmatics; https://www.geneious.com). To form the long-rigid linker, the helix-forming sequence Lys-Gln-Arg-Asp-Leu-Glu-Ala-Glu-Ala-Ala-Ala-Ala-Glu was inserted between the 1TEL and DARPin domains. In most cases, no substitutions were introduced into the DARPin, except for the single amino-acid N-terminal truncation to shorten the α-helical linker between 1TEL and the DARPin in the 10×His-1TEL-short-rigid DARPin construct. This same construct also bore a Lys-Lys→Arg-Asp substitution in its immediate N-terminus (N-terminus-LGKKLL… → N-terminus-LGRDLL….), an unexpected artefact of cloning. These substitutions never participated in crystal contacts in the two solved structures of this construct.

Similarly, fusions of 1TEL with the human thirty-eight-negative kinase-1 (TNK1) ubiquitin-associated (UBA) domain (residues 590–666; UniProt Q13470) were designed using *Foldit* (Kleffner *et al.*, 2017 ▸) and *PyMOL* (https://pymol.org/2/; Schrödinger). We replaced the Gly-Gly linker in the 1TEL-GG-UBA construct (PDB entry 7tdy; Nawarathnage *et al.*, 2023 ▸) with either a Pro-Ala dipeptide (semi-flexible), a single Lys residue (short-rigid) or a Lys-Gln-Arg-Asp-Leu-Glu hexapeptide (long-rigid). In the case of the 10×His-1TEL-short-rigid-UBA fusion, Leu591 of the UBA domain also had to be mutated to Val to resolve a steric clash. The 1TEL-GG-UBA, 1TEL-PA-UBA and 1TEL-long-rigid-UBA constructs were placed downstream of a 10×His-SUMO domain to allow His-tag removal prior to crystallization, while the 10×His-1TEL-short-rigid-UBA construct was cloned immediately downstream of a permanent 10×His tag.

Gene fragments were synthesized by Twist Bioscience (https://www.twistbioscience.com) and were assembled into the pET42_SUMO vector after cutting with XhoI (GG_*n*_ and PA_*n*_ constructs) or with both XhoI and NdeI (most constructs; Walls *et al.*, 2022 ▸) using Gibson assembly (Gibson *et al.*, 2009 ▸). The plasmids were then introduced into *Escherichia coli* BL21(DE3) cells and sequence-verified using Sanger sequencing in both directions by Eton Bioscience (https://www.etonbio.com)

### 2.2. Protein expression

Lysogeny broth (Luria–Bertani medium, LB) supplemented with 100 µg ml^−1^ kanamycin and 0.35% glucose was inoculated with colonies from transformation or with 10 µl frozen glycerol cell stock and shaken at 37°C and 250 rev min^−1^ overnight for approximately 16 h. The next day, 10 ml of the overnight culture was added to 1 l LB medium supplemented with 0.05% glucose and 50 µg ml^−1^ kanamycin and incubated at 37°C and 250 rev min^−1^. The optical density (OD) was measured every 30 min until it reached 0.8, at which point 100 µl 1 *M* isopropyl β-d-1-thiogalactopyranoside (IPTG) was added. The culture was then incubated at 18°C and 250 rev min^−1^ overnight. The following day, the cells were collected by centrifugation, snap-frozen in liquid nitrogen and stored at −80°C.

### 2.3. Purification of 1TEL-GG-DARPin, 1TEL-PA-DARPin, 1TEL-GGG-DARPin, 1TEL-PAA-DARPin, 1TEL-GG-UBA, 1TEL-PA-UBA, 1TEL-long-rigid-UBA and 3TEL-GGG-DARPin (with cleavable 10×His-SUMO tag)

All purification steps were performed on ice or in a 4°C refrigerator. The cell pellet was weighed and lysed using 3 ml wash buffer (50 m*M* Tris pH 8.8, 200 m*M* KCl, 50 m*M* imidazole) per gram of wet cell paste supplemented with 0.3 mg ml^−1^ lysozyme, 0.03 mg ml^−1^ deoxyribonuclease I, 0.2 mg ml^−1^ phenylmethylsulfonylfluoride (PMSF) and 1% methanol. The lysate was run through a homogeniser twice with a nozzle pressure of 124 MPa (NanoDeBEE 45-2, Pion Inc.). During one purification of SUMO-1TEL-PA-DARPin, cell lysis was instead accomplished via sonication at 60% power with 12 s on/59 s off for 25 cycles (Qsonica Q500) in a spinning ice bath. The resulting lysate was centrifuged at 40 000*g* and the supernatant was loaded onto 2 ml HisPure Ni–NTA resin (Thermo Scientific). The column was then washed with 10 column bed volumes (CV) of wash buffer. The column was eluted with 10 ml elution buffer (50 m*M* Tris pH 8.8, 200 m*M* KCl, 400 m*M* imidazole) until protein was no longer detected using the Bradford reagent (Bradford, 1976 ▸). Elution fractions containing significant protein were then desalted using several PD-10 desalting columns (Cytiva) in parallel, with 2.5 ml elution fraction added to each desalting column. The 10×His-SUMO tag was removed by incubating the protein (at 1.5–3.5 mg ml^−1^ in an 8–10 ml volume) overnight at 4°C with 0.5 mg SUMO protease per 100 ml protein solution (Lau *et al.*, 2018 ▸). The SUMO protease and cleaved 10×His-SUMO tags were removed by flowing the cleavage reaction over 2 ml fresh Ni–NTA resin. The protein was then concentrated to 5.5–8.7 mg ml^−1^ in a 3 ml volume (Amicon Ultra centrifugal filter, 10 kDa molecular-weight cutoff, Millipore Sigma) and loaded onto a 100 ml Superdex 200 Prep Grade size-exclusion column (Cytiva). The final purified proteins were judged to be greater than 95% pure by SDS–PAGE.

### 2.4. Purification of 10×His-1TEL-GG-DARPin, 10×His-1TEL-PA-DARPin, 10×His-1TEL-GGG-DARPin, 10×His-1TEL-short-rigid-DARPin, 10×His-1TEL-medium-rigid-DARPin, 10×His-1TEL-long-rigid-DARPin and 10×His-1TEL-short-rigid-UBA (with permanent 10×His-SUMO tag)

All steps for these constructs were identical to those described above for 10×His-SUMO-1TEL constructs, except that the SUMO tag-cleavage and tag-removal steps were omitted.

### 2.5. Crystallization

The concentrations of the purified proteins were set to 1, 9 and 18 mg ml^−1^ and they were screened against both commercial screens (PEG/Ion, SaltRx and Index, Hampton Research) and in-house custom screens (PEG Custom). 1.2 µl of each purified protein was combined with 1.2 µl of each crystallization solution in a sitting-drop format (SPT Labtech Mosquito) and equilibrated against a 50 µl reservoir of each crystallization solution via vapour diffusion. Crystals were mounted and passed through 20% glycerol in reservoir solution as a cryoprotectant before snap-freezing in liquid nitrogen.

### 2.6. Data collection, data reduction and structure solution

Crystallographic diffraction data were collected remotely on the Stanford Synchrotron Radiation Lightsource (SSRL) beamlines 9-2 and 12-2 at 100 K. Diffraction images were processed using *autoPROC* (Vonrhein *et al.*, 2011 ▸), which indexed, integrated, scaled and merged the data. The structures were solved by molecular replacement (*phenix.phaser* 1.20.1-4487; Liebschner *et al.*, 2019 ▸), using search models for 1TEL and DARPin derived from PDB entry 7n2b (Nawarathnage *et al.*, 2022 ▸) to determine the initial phases. Final refinement was performed using *phenix.refine* (Liebschner *et al.*, 2019 ▸), with iterative cycles of model building in *Coot* (Emsley *et al.*, 2010 ▸) and validation to achieve well refined structures suitable for structural analysis.

## 3. Results

### 3.1. Previous work reveals that the use of 10×His tags can impact the preferred docked configuration of the target protein against its own 1TEL polymer

In our initial 1TEL–CMG2 study (Nawarathange *et al.*, 2023 ▸), a cleavable 10×His-SUMO tag was incorporated to enhance protein solubility and facilitate purification. This approach presents a trade-off. During protein purification, the SUMO protease used to cleave the 10×His tag sometimes results in the eventual proteolytic cleavage of the target protein, which can disrupt 1TEL–target polymer–polymer association and impair crystallization. However, as observed in another prior study (Gajjar *et al.*, 2023 ▸), retention of the 10×His tag can interfere with selected docked orientations of the target protein. We postulate that in some constructs the 10×His tag can block the target-protein docking configurations necessary for efficient crystallization.

To further evaluate the impact of the 10×His tag on 1TEL polymerization and crystallization, we generated 1TEL–DARPin constructs with and without the tag. Rigid 1TEL–target linkers remove the rate-limiting step of target protein docking to its host polymer but also limit the conformational flexibility of the target protein, essentially locking it into a single docked configuration, irrespective of any His tag. Because of this, for constructs containing rigid linkers (1TEL-short-rigid-DARPin, 1TEL-medium-rigid-DARPin and 1TEL-long-rigid-DARPin), we did not create His-tag-free versions. In contrast, for constructs with flexible and semi-flexible linkers, which allow the target protein to adopt multiple conformations, we generated both His-tagged and His tag-free versions to evaluate the impact of the His tag on protein behaviour and crystallization.

### 3.2. Steric interference likely prevents 1TEL-GGG-DARPin from forming uniform 1TEL fusion polymers

**Figure 1:**
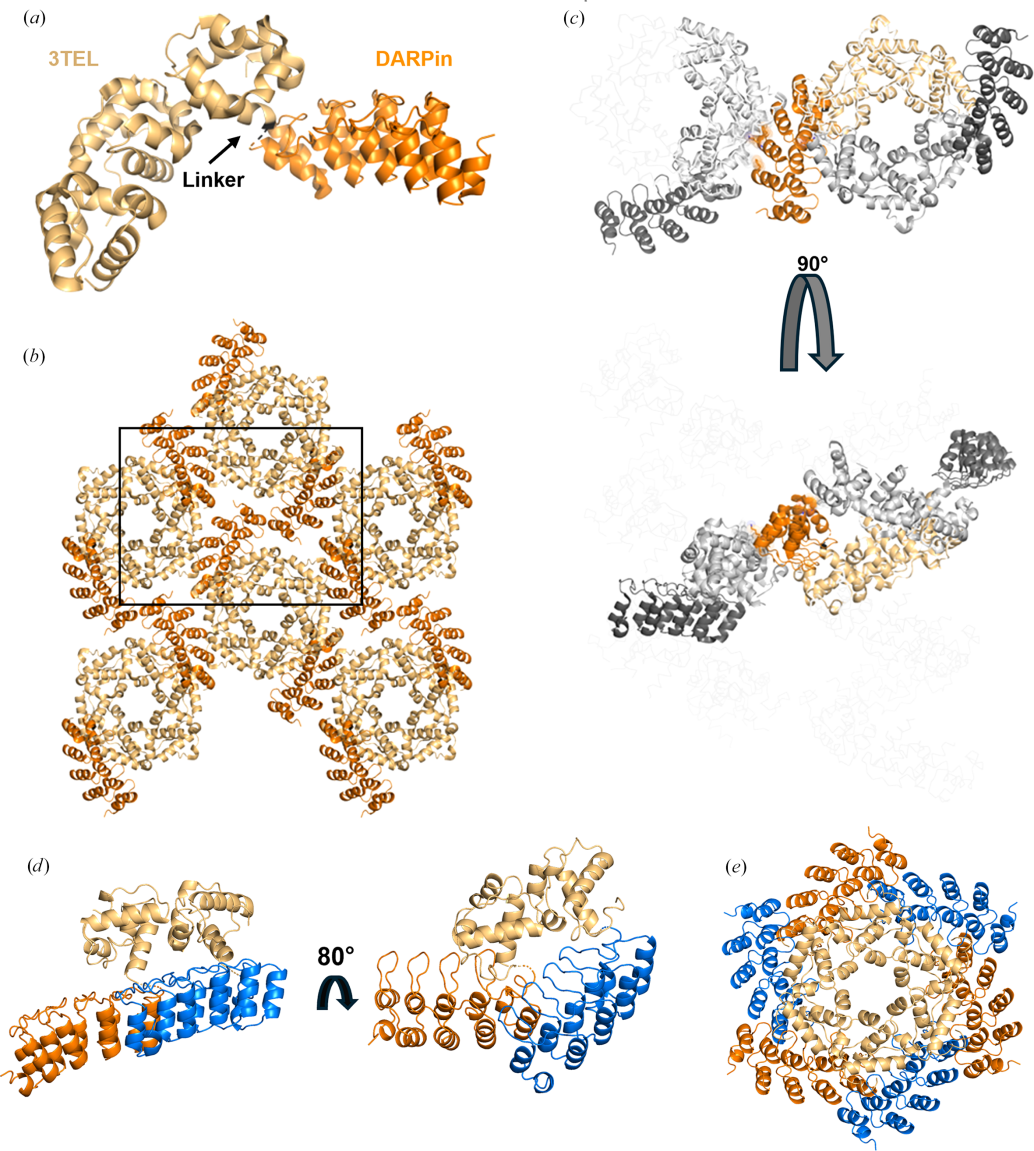
Structural and lattice interactions of 3TEL-GGG-DARPin. (*a*) Structure of the 3TEL-GGG-DARPin monomer (PDB entry 9e4q), with 3TEL shown in pale orange and DARPin in bright orange. (*b*) The 3TEL-GGG-DARPin unit cell (space group *P*2_1_2_1_2_1_) is outlined in black, with symmetry mates displayed in the same colouring as in (*a*). (*c*) Crystal lattice contacts in this crystal, with 3TEL polymers shown as ribbons and symmetry mates displayed in grey. 3TEL regions are in light grey, DARPin regions are in dark grey and the monomer is coloured as in (*a*). (*d*) The DARPin docked configuration from the 3TEL-GGG-DARPin structure superimposed onto successive 1TEL subunits of a 1TEL polymer. Neighbouring DARPins are coloured orange and blue. (*e*) As in (*d*), but for one helical turn of the canonical 1TEL *P*6_5_ polymer. Figures were prepared using *PyMOL* (Schrödinger) and Microsoft *PowerPoint*.

In our early experiments, flexible or rigid linkers in 1TEL–DARPin and 3TEL–DARPin constructs were compared. 1TEL constructs consist of a single TEL SAM domain genetically fused to the DARPin, while 3TEL constructs consist of three TEL SAM domains fused in tandem and linked by long flexible linkers between their termini (Nauli *et al.*, 2007 ▸; Poulos *et al.*, 2017 ▸). The DARPin was fused to the C-terminus of the third SAM domain. Flexible linkers consisted of Gly-Gly-Gly (GGG) and rigid linkers consisted of a continuous α-helical fusion of the last SAM domain C-terminus and the DARPin N-terminus, with 13 strongly helical amino acids (KQRDLEAEAAAAE, as reported by Nauli *et al.*, 2007 ▸) placed between the two (forming a long-rigid linker). The four constructs were as follows: 1TEL-GGG-DARPin, 1TEL-long-rigid-DARPin, 3TEL-GGG-DARPin and 3TEL-long-rigid-DARPin. All constructs gave crystals, but only 3TEL constructs [3TEL-GGG-DARPin (PDB entry 9e4q) and 3TEL-long-rigid-DARPin (PDB entry 7n2b; Nawarathnage *et al.*, 2022 ▸)] yielded crystals of sufficient quality to provide diffraction data, with resolutions of 3.58 and 3.22 Å, respectively. The complete structure of 3TEL-GGG-DARPin is reported here and shown in Figs. 1 ▸(*a*)–1 ▸(*c*) and Table 2 ▸. The structure shows that the three unique crystal contacts, all of which are DARPin–1TEL contacts, primarily rely on van der Waals interactions, with only a few hydrogen bonds. This 3TEL-GGG-DARPin structure is remarkable in that similar to 3TEL-long-rigid-DARPin it exhibits 3TEL polymers arranged in successive layers of polymers, with each layer having an N→C polymer orientation opposite to the layers before and after it. The only other published 3TEL fusion structure is PDB entry 5l0p, which did not exhibit alternating polymer orientations (Poulos *et al.*, 2017 ▸).

Since the DARPin in 3TEL-GGG-DARPin adopted a stable docked configuration against the 3TEL polymer and produced reasonable diffraction data, we questioned why 1TEL-GGG-DARPin failed to produce diffraction-quality crystals. To answer this, we superimposed the 3TEL-GGG-DARPin (PDB entry 9e4q) DARPin docked configuration onto a 1TEL polymer, as shown in Figs. 1 ▸(*d*) and 1 ▸(*e*). This analysis revealed that the DARPin docked configuration observed in the 3TEL-GGG-DARPin structure could not be accommodated if there were six copies of the DARPin per turn of a 1TEL polymer (as in 1TEL-GGG-DARPin). Had 1TEL-GGG-DARPin adopted this docked configuration, the DARPins would clash with each other, preventing them from all adopting the same docked configuration against the 1TEL polymer. This situation would prevent uniform polymer–polymer interactions and thus prevent the formation of a regular ordered crystallographic lattice required for high-quality diffraction.

### 3.3. Crystallization of 1TEL–DARPin constructs with various linkers

**Figure 2:**
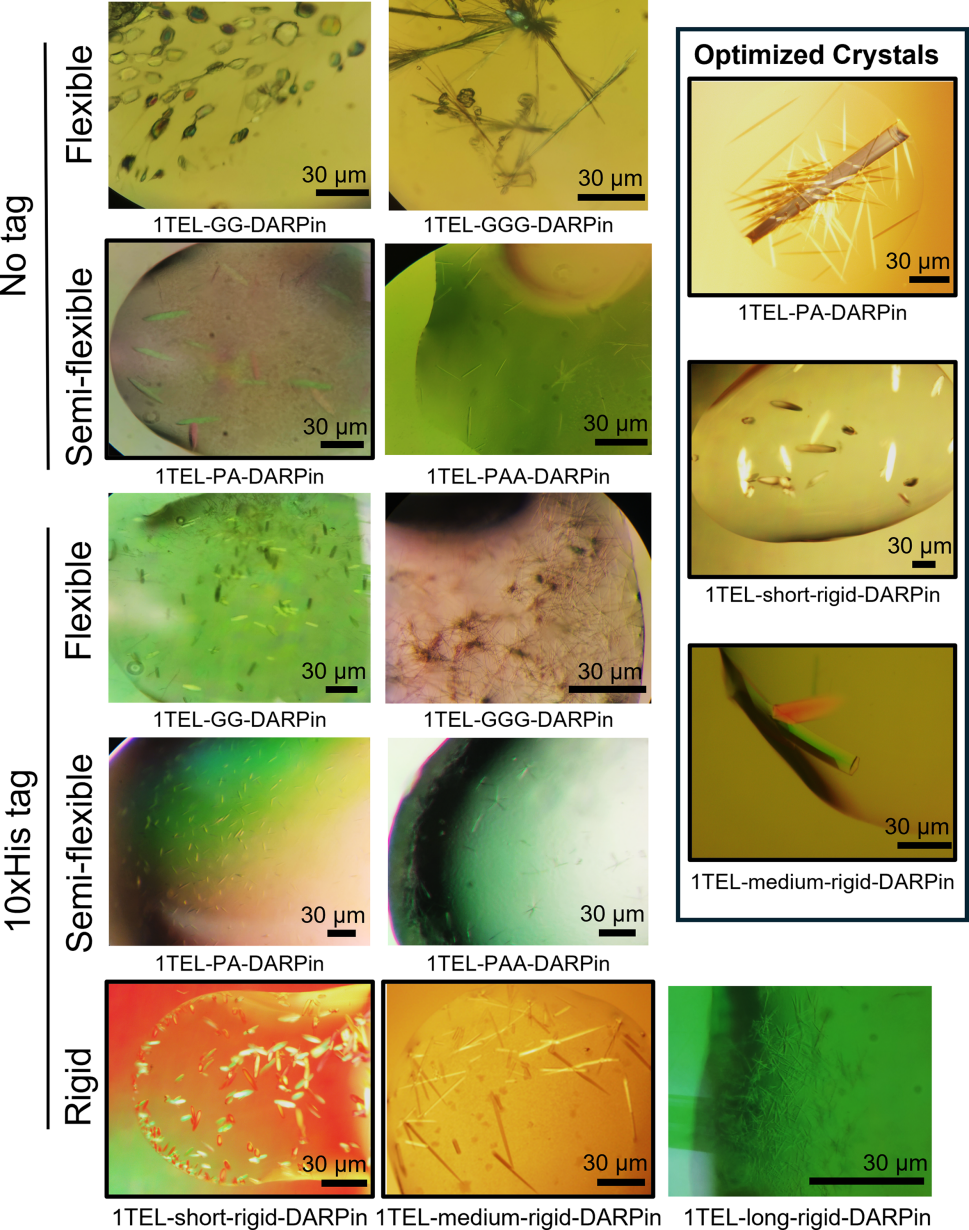
Crystals of 1TEL–DARPin constructs with flexible, semi-flexible and rigid linkers. Representative crystals obtained for all 1TEL–DARPin constructs, both with (10×His Tag) and without (No Tag) the His-tag. Constructs outlined in black represent those selected for further optimization, resulting in better quality crystals. Crystals from those optimizations are shown in the offset box at the top right. The boxed area highlights representative optimized crystals obtained from three constructs: 1TEL-PA-DARPin, 10×His-1TEL-short-rigid-DARPin and 10×His-1TEL-medium-rigid-DARPin. Images were captured using *SeBaView* (Laxco) and figures were prepared using Microsoft *PowerPoint*.

We tested flexible (GG and GGG), semi-flexible (PA and PAA) and rigid α-helical fusion (short-rigid, medium-rigid and long-rigid) linkers (Table 1 ▸). All constructs were expressed, purified and crystallized independently at least twice by two different teams of students, and all constructs yielded crystals (Table 3 ▸, Fig. 2 ▸). Overall, rigid linkers crystallized faster than semi-flexible (Pro-Ala and Pro-Ala-Ala) linkers, which in turn crystallized faster than flexible (Gly_*n*_) linkers, confirming that a rate-limiting step in crystal growth is the docking of the target protein to its host 1TEL polymer (Nawarathnage *et al.*, 2023 ▸). Some constructs gave crystals that were too small for diffraction using standard synchrotron beamlines, and others formed large crystals that nevertheless failed to diffract. This result indicates that crystal size alone is not always a predictor of diffraction quality.

Among the rigid linker constructs, the 10×His-1TEL-short-rigid-DARPin construct crystallized more rapidly than the medium-rigid and long-rigid linker constructs. Additionally, shorter rigid linkers produced larger crystals, suggesting that linker length in helical fusions may influence the quality and/or the rate of crystal packing, possibly due to residual motion afforded by the longer α-helical linkers. The short linker may have afforded less residual motion to the DARPin, allowing the 1TEL–DARPin polymers to more quickly dock against each other and thus more readily expand the growing crystal lattice along the *a* and *b* axes of the unit cell (Table 3 ▸).

Most semi-flexible 10×His-1TEL-PA-DARPin and 10×His-1TEL-PAA-DARPin constructs exhibited poor crystal morphology (pseudo-crystals) and the opposite crystallization propensity trend to that of the rigid linker constructs. While shorter rigid linker constructs produced larger crystals with shorter crystallization times, the semi-flexible linkers Pro-Ala and Pro-Ala-Ala formed equally large crystals, with those with the Pro-Ala-Ala linker appearing 2–8 times faster and diffracting to better resolution but having a moderately reduced crystallization propensity. These results show that semi-flexible linker length does not impact crystal size in this 1TEL–DARPin system but does affect crystallization time and crystal quality (Table 3 ▸).

The flexible linkers GG and GGG also behaved differently to both rigid and semi-flexible linkers. While flexible 10×His-1TEL-GG-DARPin and 10×His-1TELGGG-DARPin constructs exhibited similar crystallization speed and quality trends to the semi-flexible constructs above, the flexible linker constructs exhibited the opposite trend in crystallization propensity and a clear trend in crystal size, with the Gly-Gly linker resulting in larger crystals than the Gly-Gly-Gly linker. In spite of their smaller size, the Gly-Gly-Gly linker crystals were the only flexible-linker crystals to diffract. Crystal morphology was generally poor (needles) across all flexible constructs, which may have impaired the diffraction quality, suggesting that increased target-protein conformational freedom may impair the formation of a regular crystal lattice in this 1TEL–DARPin system (Table 3 ▸).

Removing the 10×His tag from the semi-flexible and flexible constructs significantly increased their crystallization rate, improved the crystal morphology of the shorter members of each linker type and markedly improved their crystallization propensity. While removing the 10×His tag had no significant effect on crystal size, it markedly improved the crystal quality and diffraction limits of the 1TEL-PA-DARPin construct.

Crystals obtained from screening sparse crystallization conditions diffracted to no better than 3.3–3.5 Å resolution, obscuring the positions of many side chains. We therefore sought to determine higher resolution structures of constructs with initial screening diffraction limits better than 4 Å (10×His-1TEL-short-rigid-DARPin, 10×His-1TEL-medium-rigid-DARPin and 1TEL-PA-DARPin) to investigate the structural causes of the observed crystallization trends. We further sought to determine the maximum achievable resolution for these 1TEL–DARPin constructs. Therefore, we optimized the crystallization conditions of these three constructs by more finely sampling pH and precipitant concentrations in a grid format. Table 4 ▸ summarizes selected statistics based on the crystal quality.

### 3.4. Optimizing the crystallization of initial hits from all linker types

#### 3.4.1. Condition optimization did not significantly improve the resolution of 10×His-1TEL-medium-rigid-DARPin crystals

**Figure 3:**
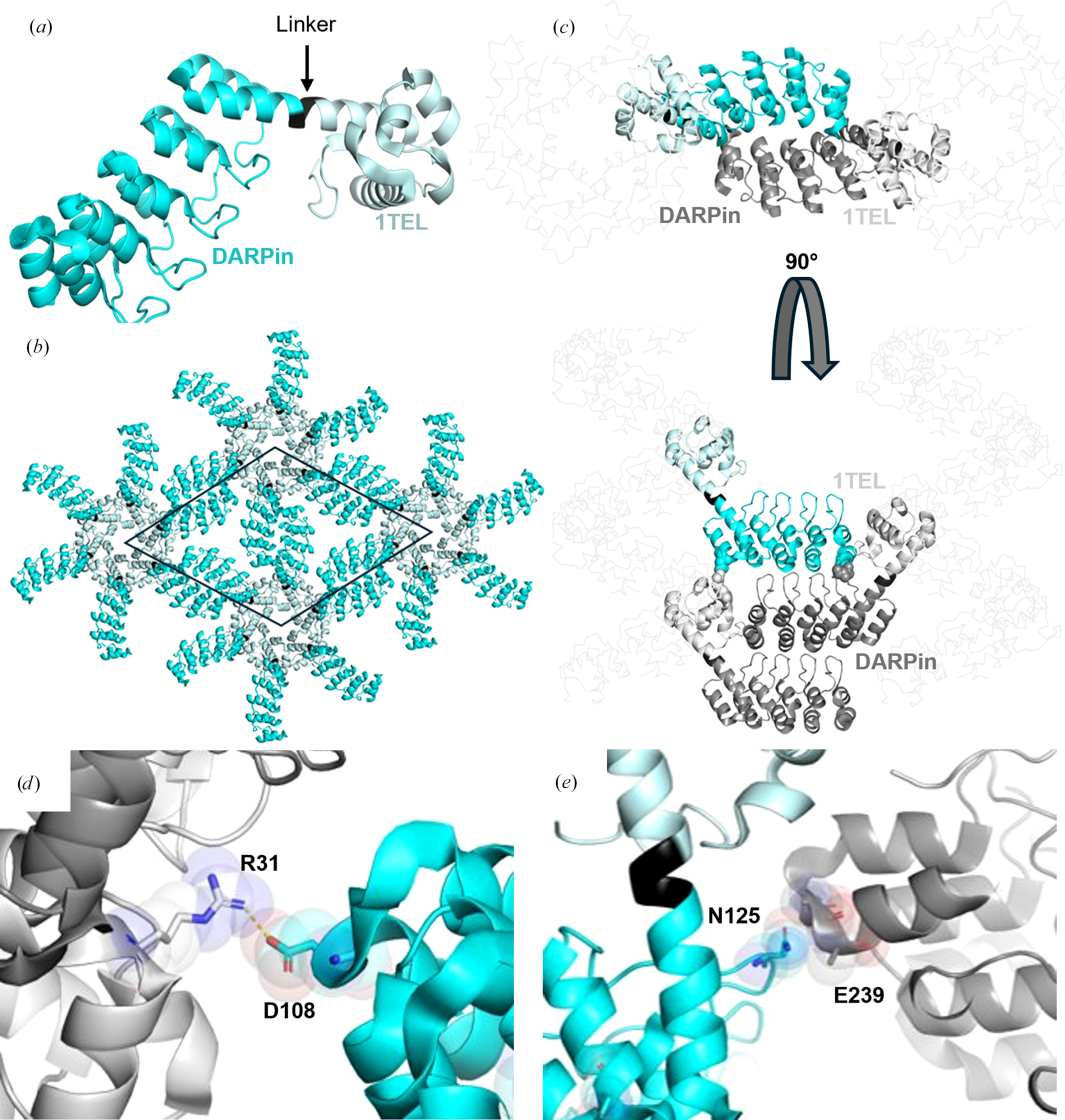
Structure and lattice interactions of a 1TEL-medium-rigid-DARPin crystal. (*a*) Structure of the 10×His-1TEL-medium-rigid-DARPin monomer (PDB entry 9dp8), with 1TEL shown in pale cyan, the medium-rigid linker in black and DARPin in bright cyan. (*b*) The unit cell of 10×His-1TEL-medium-rigid-DARPin in space group *P*6_5_ is outlined in black, with symmetry mates displayed in the same colouring as in (*a*). (*c*) Crystal lattice representation with 1TEL polymers shown as ribbons and symmetry mates displayed in grey. 1TEL regions are pastel cyan and light grey, DARPin regions are dark grey and the monomer is coloured as in (*a*). (*d*) Close-up view of the interaction between the DARPin (bright cyan) and the 1TEL unit one helical turn below it (light grey). Proteins are depicted in cartoon representation, with interacting residues shown as sticks and spheres. (*e*) As in (*d*), but showing interactions between the DARPin (bright cyan) and the adjacent DARPin from a neighbouring polymer. Figures were prepared using *PyMOL* (Schrödinger) and Microsoft *PowerPoint*.

The 10×His-1TEL-medium-rigid-DARPin originally gave crystals diffracting to an average of 3.34 Å resolution. Optimized crystals diffracted to an average of 3.28 Å. However, these latter crystals were very mosaic and did not perform well in data processing, so we solved the structure with a non-optimized crystal that diffracted to 3.47 Å resolution (Table 2 ▸, Figs. 3 ▸*a*, 3 ▸*b* and 3 ▸*c*). The medium-rigid linker essentially ‘locked’ the DARPin into a single docked configuration, and while its rigidity stabilized the protein structure, it resulted in fewer crystal contacts within the lattice relative to the other constructs (Figs. 3 ▸*d* and 3 ▸*e*, Table 5 ▸). Analysis of the crystal lattice revealed that weak interactions stabilize the DARPin target protein, which is consistent with the observed resolution. There were two crystal contacts stabilizing the DARPin, one forming a charged hydrogen bond between Arg31 of the DARPin and Asp108 of the 1TEL subunit one helical turn below it (Fig. 3 ▸*d*). The other crystal contact was a weak hydrogen bond between Asn125 of the DARPin and the backbone of Glu239 on an adjacent DARPin (Fig. 3 ▸*e*). This contact was additionally stabilized by van der Waals inter­actions and had an interface area of 157 Å^2^ (Fig. 3 ▸*e*). These sparse and relatively weak interactions are likely to contribute to the limited resolution observed with this construct.

#### 3.4.2. Optimized 10×His-1TEL-short-rigid-DARPin crystals exhibit two distinct crystal forms

**Figure 4:**
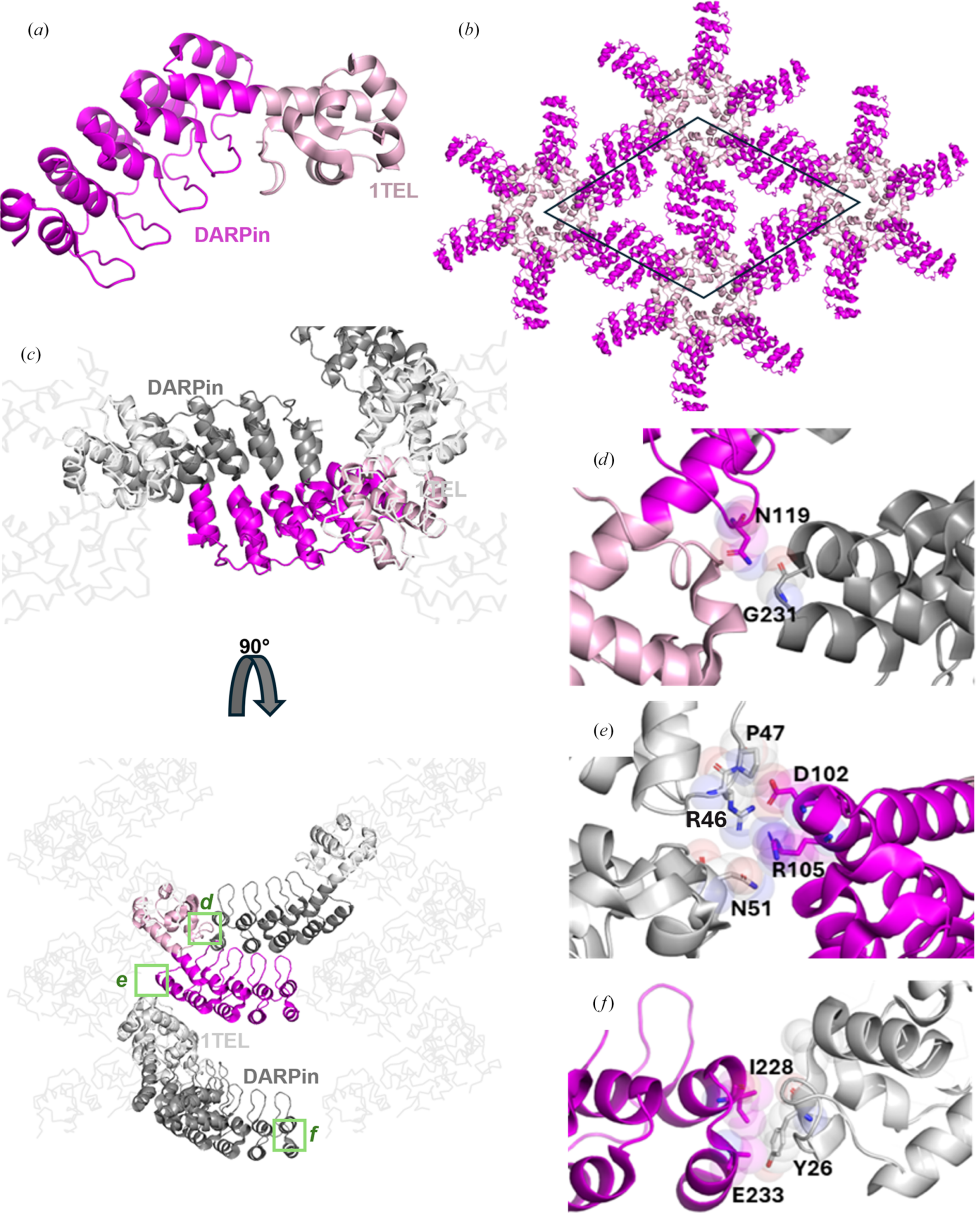
Structure and lattice interactions of a ‘2-fold’ 1TEL-short-rigid-DARPin crystal. (*a*) Structure of the ‘2-fold’ 1TEL-short-rigid-DARPin monomer (PDB entry 9dvg), with 1TEL shown in pale pink and DARPin in magenta. (*b*) The unit cell of 1TEL-short-rigid-DARPin in space group *P*6_5_ is outlined in black, with symmetry mates displayed in the same colouring as in (*a*). (*c*) Crystal lattice representation with 1TEL polymers shown as ribbons and symmetry mates displayed in grey. 1TEL regions are in light grey, DARPin regions are in dark grey and the monomer is coloured as in (*a*). (*d*) Close-up view of the interaction between two DARPins, with interacting residues shown as sticks and spheres. (*e*, *f*) Two unique interactions between the DARPin (magenta) and nearby 1TELs (light grey) from the host polymer or a neighbouring polymer. Figures were prepared using *PyMOL* (Schrödinger) and Microsoft *PowerPoint*.

We observed two distinct crystal forms for the 10×His-1TEL-short-rigid-DARPin construct, which we will refer to hereafter as the ‘2-fold’ and ‘3-fold’ crystal forms, respectively. The ‘2-fold’ crystal form showed DARPin–DARPin crystal contacts intersecting the twofold screw axes of the *P*6_5_ unit cell (Figs. 4 ▸*a*, 4 ▸*b* and 4 ▸*c*), similar to those observed in the 10×His-1TEL-medium-rigid-DARPin construct, while the ‘3-fold’ lattice revealed a novel arrangement in which the DARPins came together in a staircase-like formation intersecting the *P*3_2_ screw axes of the *P*6_5_ unit cell. When viewed along the *c* axis of the unit cell, three DARPins meet in a triangular arrangement. The ‘3-fold’ lattice featured more crystal contacts, showed higher internal order and achieved a higher resolution compared with the ‘2-fold’ lattice. The difference between these packing arrangements is important because in the ‘2-fold’ packing arrangement each DARPin only contacted DARPins originating from one other 1TEL polymer, while in the ‘3-fold’ packing arrangement each DARPin contacted DARPins originating from two distinct 1TEL polymers.

#### 3.4.3. The 10×His-1TEL-short-rigid-DARPin ’2-fold’ crystal form resembles the medium-rigid crystal form, with relatively weak contacts

We solved the structure of a ‘2-fold’ 10×His-1TEL-short-rigid-DARPin crystal at 3.54 Å resolution (Table 2 ▸; PDB entry 9dvg). The structure exhibits a similar lattice architecture to the 10×His-1TEL-medium-rigid-DARPin construct (Fig. 3 ▸*a*, PDB entry 9dvg), although with distinct crystal contacts. Analysis of this structure revealed three key interaction points stabilizing the crystal lattice. The first was a DARPin–DARPin crystal contact stabilized by a hydrogen bond between the side-chain amide N atom of Asn119 of one DARPin and the backbone carbonyl O atom of another (Fig. 4 ▸*d*). The remaining two contacts were DARPin–1TEL interactions. One included van der Waals interactions and a salt bridge between the side chain of Asp102 of a DARPin and the side chain of Arg46 of the 1TEL subunit one helical turn below it in the same 1TEL polymer (Fig. 4 ▸*e*). The last contact is another weak van der Waals interaction between the side chains of Ile228 and Glu233 of a DARPin and Tyr26 of the 1TEL subunit of a neighbouring 1TEL polymer (Fig. 4 ▸*f*). The total interface area was 307 Å^2^. The weaker nature of these limited interactions also likely contributed to the lower resolution achieved with this construct.

#### 3.4.4. The ’3-fold’ crystal form of 10×His-1TEL-short-rigid-DARPin exhibits enhanced crystal contacts

**Figure 5:**
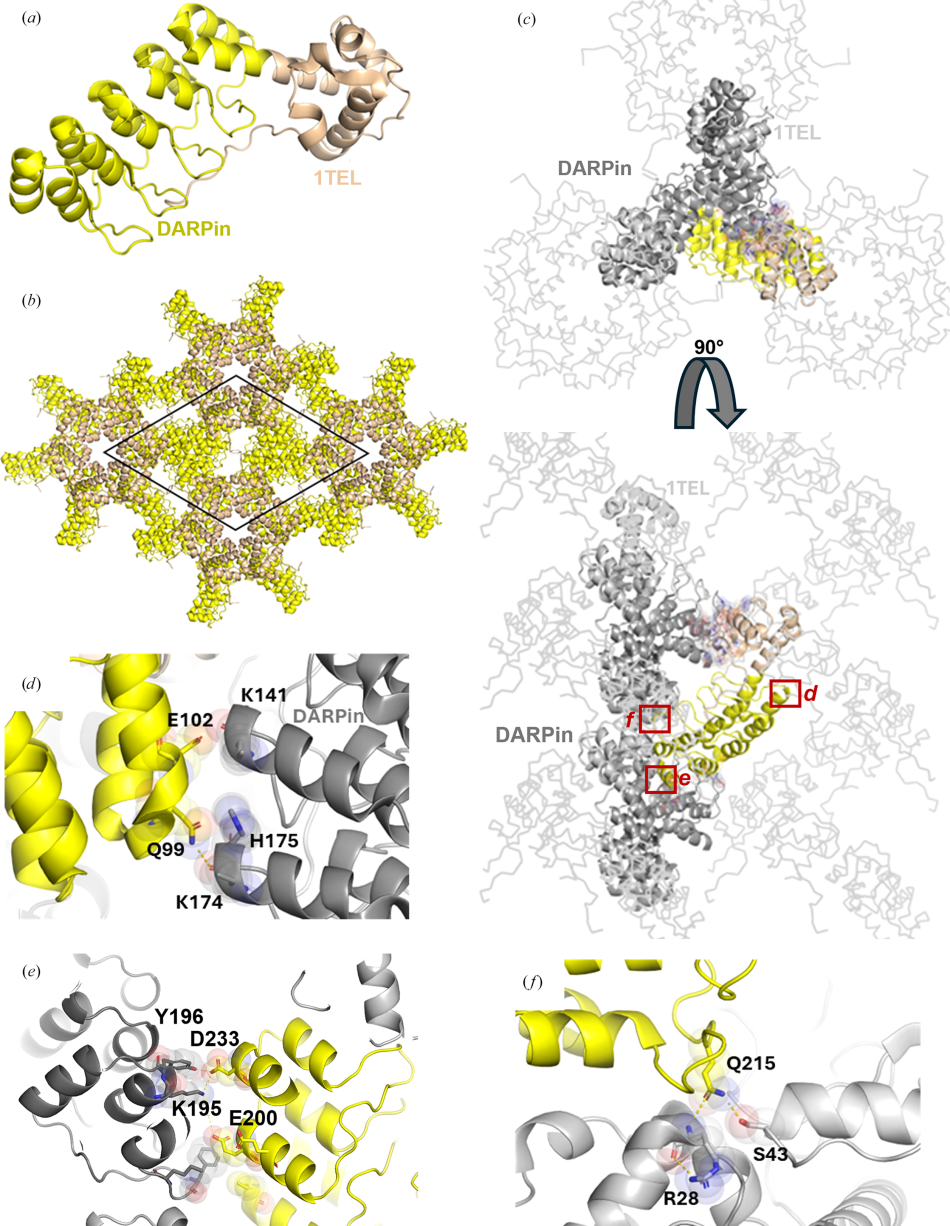
Structure and lattice interactions of a ‘3-fold’ 1TEL-short-rigid-DARPin crystal. (*a*) Structure of the ‘3-fold’ 10×His-1TEL-short-rigid-DARPin monomer (PDB entry 9znb), with 1TEL shown in wheat and DARPin in yellow. (*b*) The unit cell of ‘3-fold’ 10×His-1TEL-short-rigid-DARPin in space group *P*6_5_ is outlined in black, with symmetry mates displayed in the same colouring as in (*a*). (*c*) Crystal lattice representation with 1TEL polymers shown as ribbons and symmetry mates displayed in grey. 1TEL regions are in light grey, DARPin regions are in dark grey and the monomer is coloured as in (*a*). (*d*, *e*) Close-up view of the interactions between the DARPin (yellow) and the adjacent DARPins from neighbouring polymers (dark grey). Proteins are depicted in cartoon representation, with interacting residues shown as sticks and spheres. (*f*) As in (*d*) and (*e*), but showing interactions between the DARPin (yellow) and a 1TEL subunit (light grey) one helical turn above it in the same 1TEL polymer. Also shown are salt-bridge contacts, represented by yellow dotted lines. Figures were prepared using *PyMOL* (Schrödinger) and Microsoft *PowerPoint*.

We solved the structure of a ‘3-fold’ 10×His-1TEL-short-rigid-DARPin crystal at 1.77 Å resolution (Table 2 ▸, Figs. 5 ▸*a*, 5 ▸*b* and 5 ▸*c*; PDB entry 9znb). In this structure we found a unique staircase-like structure along the *P*3_2_ axes of the unit cell, where DARPins assembled in a threefold helical arrangement. This crystal achieved a better resolution, likely due to enhanced crystal contacts. Unlike the ‘2-fold’ crystal form, the ‘3-fold’ crystal form featured two unique DARPin–DARPin crystal contacts per asymmetric unit, each involving more residues and stronger interactions, including some salt bridges. The first contact involves a hydrogen bond between Gln99 of the DARPin and a backbone carbonyl of Lys174 of a neighbouring DARPin (Fig. 5 ▸*d*). The second contact included a salt bridge between Glu200 of the DARPin and Lys195 of a neighbouring DARPin subunit, and a hydrogen bond between Asp233 of the DARPin and Lys195 of a neighbouring DARPin stabilized by van der Waals interactions (Fig. 5 ▸*e*). The final interaction consisted of two hydrogen bonds between Gln215 of the DARPin and the side chain of Ser43 and the backbone amide of Arg28, both from a neighbouring 1TEL subunit from another 1TEL polymer (Fig. 5 ▸*f*). The total interface area of all three interactions was 1085 Å^2^. These extensive interactions are likely to be responsible for the increased internal order and higher resolution observed with this construct.

#### 3.4.5. 1TEL-PA-DARPin achieved the highest resolution of all constructs and exhibited the most crystal contacts

**Figure 6:**
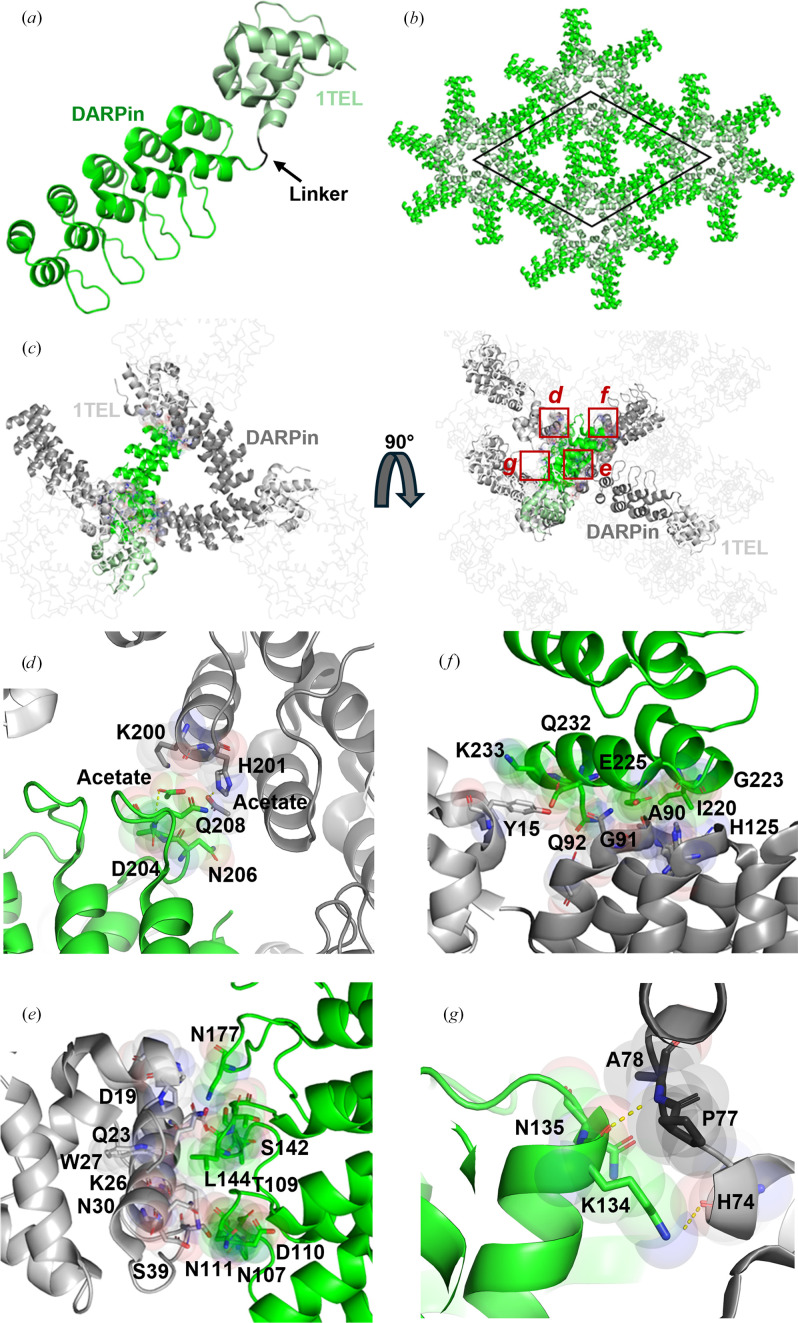
Structure and lattice interactions of a 1TEL-PA-DARPin crystal. (*a*) Structure of the 1TEL-PA-DARPin monomer (PDB entry 9db5), with 1TEL shown in pale green and the DARPin in bright green. (*b*) The unit cell of 1TEL-PA-DARPin in space group *P*6_5_ is outlined in black, with symmetry mates displayed in the same colouring as in (*a*). (*c*) Crystal lattice representation with 1TEL polymers shown as ribbons and symmetry mates displayed in grey. 1TEL regions are in light grey, DARPin regions are in dark grey and the monomer is coloured as in (*a*). (*d*)–(*g*) Close-up views of the interactions between the DARPin (bright green) and adjacent DARPin proteins from neighbouring polymers or neighbouring 1TEL units (light grey). Proteins are depicted in cartoon representation, with interacting residues shown as sticks and spheres and hydrogen bonds shown as yellow dotted lines. Figures were prepared using *PyMOL* (Schrödinger) and Microsoft *PowerPoint*.

The 1TEL-PA-DARPin construct produced the highest resolution structure in this study at 1.57 Å (Table 2 ▸; PDB entry 9db5), slightly better than the resolution of the same DARPin crystallized on its own (1.60 Å; PDB entry 4j7w; Seeger *et al.*, 2013 ▸), and exhibited strong crystal contacts (Figs. 6 ▸*a*, 6 ▸*b* and 6 ▸*c*). We observed rapid crystal dissolution during harvesting (explained below in Section 3.4.6), but were able to harvest several crystals quickly enough to outpace complete dissolution. Unlike the rigid linker constructs, this construct lacked a 10×His tag during crystallization. The DARPin was observed to form four unique crystal contacts per asymmetric unit (Fig. 6 ▸*c*). The first involved van der Waals contacts between Asn206 of the DARPin and a bridging acetate ion, which was hydrogen-bonded to His201 of a neighbouring DARPin. A second acetate ion made van der Waals contact with Asn206 of the DARPin and with Lys200 of the neighbouring DARPin, as well as a weak hydrogen bond to the backbone of Gln208 of the DARPin, with the entire interface having an area of 162 Å^2^. In addition, Lys200 of the neighbouring DARPin formed a salt bridge to Asp204 of the DARPin, while Lys167 formed salt bridges to both Asp217 and the C-terminal carboxylate of Asn235 of the DARPin (Fig. 6 ▸*d*). The second contact occurred between the long finger loops of the DARPin and an adjacent 1TEL subunit. This contact consisted largely of hydrogen bonds between Asn177, Leu144, Ser142, Asn111, Asp110 and Asn107 of the DARPin and Gln23, Asp19, Ser39, Lys26 and Asn30 of the 1TEL subunit. In addition, Thr109 and Leu144 of the DARPin made van der Waals contact with Ser39 and Trp27 of the 1TEL subunit, with the entire interface having an area of 470 Å^2^ (Fig. 6 ▸*e*). The third contact included DARPin–DARPin van der Waals interactions between Lys233 and Tyr15, Gln232 and Gln92, Glu225 and Gly91, Gly223 and His125, and Ile220 and Ala90, all listed with residues from the DARPin first followed by the neighbouring DARPin amino acids. Also present was a salt bridge between Glu225 of the DARPin and His125 of a neighbouring DARPin, as well as hydrogen bonds between Glu225 and Gln232 of the DARPin and Gln92 and Tyr15 of a neighbouring DARPin (Fig. 6 ▸*f*), with an interface area of 420 Å^2^. The final contact involved two hydrogen bonds between Lys134 of the DARPin and residues His74, Pro77 and Ala78 of a neighbouring 1TEL (Fig. 6 ▸*g*). The total interface area was 1855 Å^2^, the greatest observed among all the constructs. Indeed, the resolution of these 1TEL–DARPin constructs correlated very well with the number and area of the unique crystal contacts made by the DARPins in each case (Table 5 ▸).

#### 3.4.6. 1TEL-PA-DARPin crystals likely dissolved due to a pH shift caused by acetic acid evaporation

During crystal harvesting, we observed that the 1TEL-PA-DARPin crystals immediately began dissolving once the well was opened. From the structure, we noted that many crystal contacts consisted of side-chain carboxylic acids hydrogen bonding to each other. While direct carboxylic acid–carboxylic acid hydrogen bonds are unusual in biological systems, they were possible in this pH 4.6 crystallization drop as a significant number of these carboxylates (p*K*_a_ values around pH 4) would be protonated at pH 4.6. The juxtaposition of these carboxylic acids likely also raised their p*K*_a_ values above 4. Numerous acetates were also visible in the electron density. Upon opening the well for crystal harvesting, acetic acid molecules undoubtedly began evaporating, shifting the equilibrium of the acetates remaining in solution toward the deprotonated state and increasing the pH of the crystal drop. This increase in pH likely began deprotonating the carboxylic acid side chains, causing them to repel each other and disrupt the corresponding crystal contacts, resulting in rapid crystal dissolution. As few 1TEL fusion crystals appear in crystallization conditions containing volatile buffer components and even fewer involve carboxylic acid–carboxylic acid hydrogen bonds, we expect that this phenomenon will not be typical of 1TEL fusion crystals.

## 4. Generalizability of linker optimization to 1TEL–UBA crystallization

**Figure 7:**
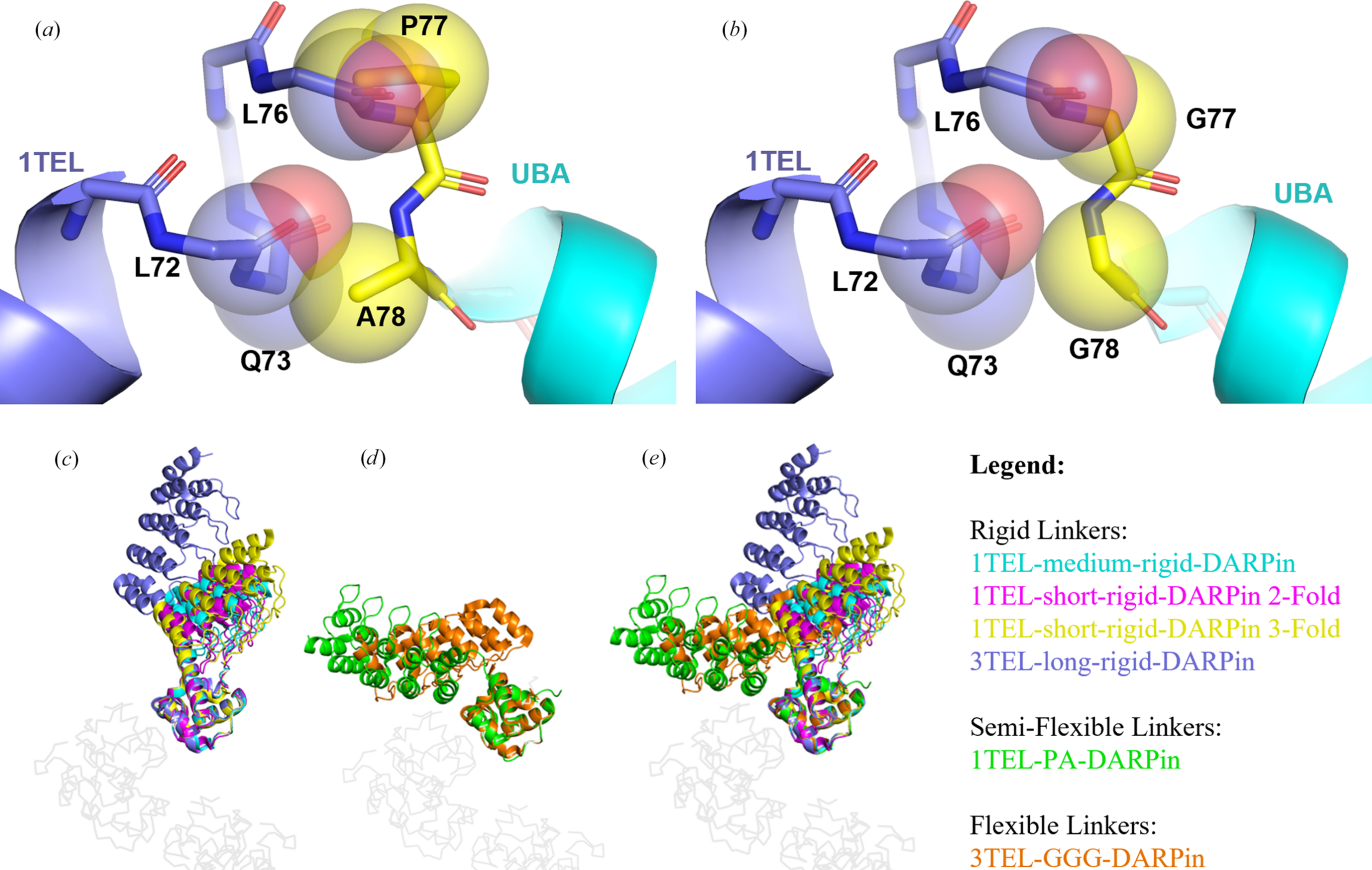
Analysis of 1TEL–UBA linkers and superposition of rigid, semi-flexible and flexible 1TEL–DARPin and 3TEL–DARPin fusions. (*a*) Superposition of a theoretical Pro-Ala linker between a 1TEL (slate) and a UBA (cyan) from the published structure of 1TEL-GG-UBA (PDB entry 7tdy) with clashes shown in spheres. (*b*) As in (*a*), but for the published PDB entry 7tdy with its original Gly-Gly linker. (*c*) Structures of constructs with rigid linkers: 1TEL-medium rigid-DARPin (PDB entry 9dp8, cyan), ‘3-fold’ 1TEL-short-rigid-DARPin (PDB entry 9znb, yellow), ‘2-fold’ 1TEL-short-rigid-DARPin (PDB entry 9dvg, pink) and 3TEL-long-rigid-DARPin (PDB entry 7n2b, blue), superimposed (via the C^α^ atoms of their 1TEL domains) onto a 1TEL polymer (light grey). (*d*) Structures of constructs with semi-flexible and flexible linkers, 1TEL-PA-DARPin (PDB entry 9db5, green) and 3TEL-GGG-DARPin (PDB entry 9e4q, orange), superimposed (via the C^α^ atoms of their C-terminal-most 1TEL domain) onto a 1TEL polymer (light grey). (*e*) Superposition of (*c*) and (*d*). Figures were prepared using *PyMOL* (Schrödinger) and Microsoft *PowerPoint*.

After determining that the semi-flexible Pro-Ala and short-rigid linkers were most effective for the 1TEL–DARPin system, we sought to validate them against a second protein target. We selected the ubiquitin-associated (UBA) domain of human thirty-eight-negative kinase 1 (TNK1) as we have previously characterized the 1TEL-GG-UBA fusion and because the UBA domain also has an α-helical N-terminus. We also report results from a previous 1TEL-long-rigid-UBA construct. We previously reported the structure of this same UBA domain at a resolution of 1.53 Å using 1TEL fusion with a Gly-Gly linker (PDB entry 7n2b; Nawarathnage *et al.*, 2023 ▸). We thus tested linking the UBA domain to 1TEL with four distinct linkers: Gly-Gly (GG; positive control), both short and long direct helical fusions (short-rigid and long-rigid) and Pro-Ala (PA) (Table 1 ▸). The 1TEL-GG-UBA, 1TEL-PA-UBA and 1TEL-short-rigid-UBA constructs successfully yielded crystals, while the 1TEL-long-rigid-UBA construct did not (Table 6 ▸). Only 1TEL-GG-UBA crystals diffracted X-rays, while crystals of constructs utilizing the short-rigid and PA linkers did not diffract. The PA linker performed particularly poorly in the 1TEL–UBA system, exhibiting poor solubility during purification. In addition, the rapid formation of hazy unstructured precipitate in roughly 90% of drops in the crystal trays was observed with 1TEL-PA-UBA and may have outpaced the formation of regularly aligned 1TEL polymers. This rapid precipitation was not seen with otherwise identical 1TEL–UBA constructs having short-rigid or Gly-Gly linkers or in any of the 1TEL–DARPin constructs at the same protein concentrations, which showed thicker, more opaque precipitate in roughly 50% of the drops. This linker-induced solubility reduction warrants future investigation. While the crystals of the ‘PA’ construct were of adequate size and exhibited a hexagonal prism crystal habit, crystals of the ‘short-rigid’ construct were too small for diffraction using standard beamlines. This indicated that the Gly-Gly linker remains the current best choice for 1TEL–UBA fusion crystallization. Diffraction statistics for this ‘GG’ construct are given at the bottom of Table 4 ▸. Notably, analysis of our previous 1TEL-GG-UBA structure reveals that a Pro-Ala linker could not permit the same UBA docked configuration to its host 1TEL polymer due to steric interference, as discussed below (Figs. 7 ▸*a* and 7 ▸*b*).

## 5. Discussion

The choice of linker between 1TEL and the target protein appears to be dependent on the target protein itself in a non-intuitive manner. The Gly-Gly linker outperformed the short-rigid and Pro-Ala linkers in the context of the 1TEL–UBA system, in stark contrast to the 1TEL–DARPin system, where Pro-Ala, Pro-Ala-Ala and short-rigid linkers performed better than Gly-Gly and Gly-Gly-Gly linkers. We propose that this differential behaviour is due to the differing sizes of the UBA (9 kDa) and DARPin (17 kDa) target proteins. Gly-Gly and Gly-Gly-Gly linkers appear to work best by enabling the target protein to fold back against its host polymer, as exhibited by the 1TEL-GG-UBA and 3TEL-GGG-DARPin crystal structures. While the UBA could fold back to lie flat against its host polymer when fused to 1TEL, the DARPin was too large to do so when fused to 1TEL (Figs. 1 ▸*d* and 1 ▸*e*). The DARPin could, however, fold back against its host polymer if only two DARPins were displayed, 180° apart, as in 3TEL-GGG-DARPin (PDB entry 9e4q). The Pro-Ala linker may have been inflexible enough to allow the DARPin docked configuration observed in the 1TEL-PA-DARPin crystal structure but not so rigid as to incur the challenges observed with the rigid α-helical 1TEL-DARPin fusions described in this study. Modelling a Pro-Ala linker in place of the Gly-Gly linker in our published 1TEL-GG-UBA structure (*PyMOL* mutagenesis tool, no minimization; PDB entry 7tdy; Nawarathnage *et al.*, 2023 ▸) reveals that a proline cannot access the Ramachandran angle of the first Gly77 of the Gly-Gly linker without incurring serious steric clashes with the backbone carbonyl of the Leu76 preceding it (Figs. 7 ▸*a* and 7 ▸*b*). Likewise, substituting an Ala for the second Gly78 in this linker results in serious clashes with the backbone carbonyl of Leu72 and Gln73 of the 1TEL domain. In fact, no other amino acids except Gly can be substituted at either of these positions in the solved 1TEL-GG-UBA structure. As the amino acids before and after the Gly-Gly linker are part of α-helices, it is also unlikely that a Pro could have been accommodated at any other position near the 1TEL–UBA junction point (Figs. 7 ▸*a* and 7 ▸*b*). When designing this fusion, we anticipated that the Pro-Ala linker would force the UBA domain to adopt a new and distinct docked orientation. Since the 1TEL-PA-UBA construct did form crystals, the UBA likely did adopt one or more new docked configurations, but possibly configurations that were not compatible with sufficiently productive or regular crystal contacts.

We also observed that the 10×His-1TEL-medium-rigid-DARPin and the ‘2-fold’ 10×His-1TEL-short-rigid-DARPin crystals exhibited similar DARPin orientations, resulting in fewer and weaker crystal contacts and likely contributing to the observed poor resolution. In contrast, the ‘3-fold’ 10×His-1TEL-short-rigid-DARPin crystal form showed improved crystal contacts and better resolution. These findings suggest that while rigid linkers may reduce the entropic cost of crystal formation by essentially ‘locking’ target proteins in place, they sometimes struggle to produce high-quality structures. By restricting the conformational flexibility of the DARPin, rigid linkers prevented the DARPin from sampling alternative docked configurations against its host 1TEL polymer. While rigid linkers eliminate the rate-limiting target protein-docking step otherwise required for polymer–polymer association and crystal growth, they may also hinder the formation of optimal crystal contacts, contributing to the formation of lower quality crystals and reduced resolution, or altogether preventing crystal formation. We hypothesize that the target protein docked configuration (to its host 1TEL/3TEL polymer) observed in solved structures using flexible and semi-flexible linkers is not necessarily the lowest energy docked configuration, but rather a docked configuration that enables productive polymer–polymer crystal contacts. These crystal contacts then contribute to improved internal order within the crystal, leading to higher resolution. Rigidly fused target proteins have no opportunity to sample multiple docked configurations in search of one that gives productive crystal contacts and so exhibit a lower (but nonzero) probability of forming strong polymer–polymer contacts. In support of this hypothesis, crystals of the 10×His-1TEL-medium-rigid-DARPin construct from this study exhibited poor diffraction limits even after crystallization-condition optimization, while the 1TEL-long-rigid-UBA fusion did not form crystals. Our previous 3TEL-long-rigid-DARPin fusion (PDB entry 7n2b) formed only thin plates, exhibiting a clear defect in target protein–target protein crystal contacts (Nawarathnage *et al.*, 2022 ▸). In a related study, we designed a 1TEL-short-rigid-claudin1-variant fusion. This construct formed small needle crystals that failed to diffract X-rays, suggesting a defect in polymer–polymer association. Additionally, in the hands of different teams of students, both the 1TEL-short-rigid-UBA fusion (from this study) and a 1TEL-short-rigid-vacuolar protein sorting-34 fusion only crystallized in nonpolymer crystal forms (to be described elsewhere). Of note is the fact that relative to the host 1TEL, the position of the DARPin domain between the ‘3-fold’ and ‘2-fold’ 10×His-1TEL-short-rigid-DARPin structures (PDB entries 9dvg and 9znb) differs by as much as 19.3 Å at the distal end of the DARPin, due to some flexibility in the conformation of the helical linker (specifically residues Leu87-Leu88). This observation confirms that even ‘rigid’ α-helical fusions retain some degree of flexibility, as previously posited (Nawarathnage *et al.*, 2022 ▸; Fig. 7 ▸*c*). This subtle displacement of the DARPin likely contributed to the differing crystal packing between the ‘2-fold’ and ‘3-fold’ 1TEL-short-rigid-DARPin structures. Superpositions of all rigid (Fig. 7 ▸*c*), flexible and semi-flexible linkers (Fig. 7 ▸*d*) and of all DARPin constructs together (Fig. 7 ▸*e*) highlight the impact of linker composition on DARPin orientation, docking, crystal contacts and overall crystal formation.

In this study, the semi-flexible PA linker produced the highest resolution and most ordered crystals for the 1TEL–DARPin system, followed closely by the short-rigid linker. However, the Gly-Gly linker remained the most effective for the 1TEL–UBA system, confirming that optimal linker choice is dependent on the target protein. These findings support the selection of a linker that provides sufficient flexibility for the target protein to adopt a docking conformation to its host polymer capable of supporting productive inter-polymer contacts. At this juncture, we recommend prioritizing short flexible or semi-flexible linkers, such as Gly-Gly or Pro-Ala, over rigid linkers to maximize the odds of productive crystal contact formation. We further recommend that rigid linkers (due to their tendency to limit crystal quality and diffraction resolution) be reserved for cases where more flexible linkers have failed. Finally, we recommend removing N-terminal polyhistidine tags from flexibly or semi-flexibly fused constructs.

## Supplementary Material

PDB reference: DARPin fused to 1TEL via a Pro-Ala linker, 9db5

PDB reference: DARPin fused to 1TEL via a helical Lys-Gln-Arg linker, 9dp8

PDB reference: DARPin fused to 1TEL via a direct helical fusion, 9dvg

PDB reference: DARPin fused to 3TEL via a Gly-Gly-Gly fusion, 9e4q

PDB reference: DARPin fused to 1TEL via a direct helical fusion in a ‘3-fold’ crystal form, 9znb

## Figures and Tables

**Table 1:** Abbreviations of all linker types Abbreviations used throughout this study. Italicized text represents the 1TEL unit, followed by three dots indicating the intervening 1TEL sequence and then by capitalized C-terminal amino acids of 1TEL. A dash separates the 1TEL sequence from the linker amino acids (bold and underlined), which are then followed by another dash and the capitalized N-terminal amino acids of the DARPin or TNK1 UBA domain; three dots then indicate the intervening DARPin or UBA sequence. Linkers used for both 1TEL and 3TEL constructs are marked with an asterisk (*).

Sequence	Name	Abbreviation
*1TEL*...HIL–LG...*DARPin*	Short-Rigid	short-rigid
*1TEL*...HIL–**KQR**–DLG...*DARPin*	Medium-Rigid	medium-rigid
*1TEL*...HIL–**KQRDLEAEAAAAE**–DLG...DARPin*	Long-Rigid	long-rigid
*1TEL*...HIL–**PA**–DLG...*DARPin*	Pro-Ala	PA
*1TEL*...HIL–**PAA**–DLG...*DARPin*	Pro-Ala-Ala	PAA
*1TEL*...HIL–**GG**–DLG*...DARPin*	Gly-Gly	GG
*1TEL*...HIL–**GGG**–DLG...DARPin*	Gly-Gly-Gly	GGG
*1TEL*...HIL–**GG**–ELQ...*UBA*	Gly-Gly	GG
*1TEL*...HIL–**PA**–ELQ...*UBA*	Pro-Ala	PA
*1TEL*...HIL–**K**–EVQ...*UBA*	Short-Rigid	short-rigid
*1TEL*...HIL–**KQRDLE**–ELQ...*UBA*	Long-Rigid	long-rigid

**Table 2:** Data-processing and refinement statistics for all structures reported in this study Values in parentheses are for the outer shell. Resolution cutoffs were determined by *autoPROC* (Vonrhein *et al.*, 2011 ▸) as the maximal resolution with a CC_1/2_ > 0.3000 and an *I*/σ(*I*) > 0.00 for the outer shell and all shells before it. ND, not used.

	3TEL-GGG-DARPin (PDB entry 9e4q)	10×His-1TEL-medium-rigid-DARPin (PDB entry 9dp8)	10×His-1TEL-short-rigid-DARPin, ‘2-fold’ crystal form (PDB entry 9dvg)	10×His-1TEL-short-rigid-DARPin, ‘3-fold’ crystal form (PDB entry 9znb)	1TEL-PA-DARPin (PDB entry 9db5)
Wavelength (Å)	0.979460	0.979460	0.979460	0.979460	0.979460
Resolution range (Å)	42.75–3.58 (3.71–3.58)	45.89–3.47 (3.60–3.47)	43.93–3.54 (3.67–3.54)	41.64–1.77 (1.84–1.77)	48.87–1.57 (1.63–1.57)
Space group	*P*2_1_2_1_2_1_	*P*6_5_	*P*6_5_	*P*6_5_	*P*6_5_
*a*, *b*, *c* (Å)	46.14, 85.5, 120.68	105.98, 105.98, 51.14	101.46, 101.46, 48.08	83.29, 83.29, 58.43	97.73, 97.73, 45.82
α, β, γ (°)	90, 90, 90	90, 90, 120	90, 90, 120	90, 90, 120	90, 90, 120
Total reflections	75782 (6751)	37143 (3892)	73291 (6217)	430714 (43478)	452015 (49961)
Unique reflections	6006 (586)	3961 (320)	3560 (359)	21254 (1434)	33052 (3468)
Multiplicity	12.6 (11.5)	9.4 (9.2)	20.6 (17.3)	20.3 (19.6)	13.7 (14.3)
Completeness (%)	99.57 (98.82)	87.47 (75.29)	99.92 (100.00)	90.37 (64.57)	94.10 (99.54)
Mean *I*/σ(*I*)	8.55 (2.36)	7.03 (0.50)	8.12 (1.86)	13.52 (0.54)	12.34 (1.38)
Wilson *B* factor (Å^2^)	80.19	147.09	135.55	31.01	17.08
*R*_merge_	0.2825 (1.132)	0.1269 (3.200)	0.1917 (2.487)	0.1493 (6.504)	0.2162 (4.199)
*R*_meas_	0.2945 (1.187)	0.1343 (3.390)	0.1966 (2.562)	0.1531 (6.676)	0.2246 (4.353)
*R*_p.i.m._	0.08171 (0.3483)	0.04332 (1.111)	0.04311 (0.6086)	0.03350 (1.490)	0.05997 (1.140)
CC_1/2_	0.993 (0.828)	0.999 (0.578)	0.999 (0.407)	0.998 (0.340)	0.996 (0.501)
CC*	0.998 (0.952)	1 (0.856)	1 (0.761)	1 (0.713)	0.999 (0.817)
Reflections used in refinement	5999 (585)	3806 (320)	3559 (359)	20369 (1434)	32999 (3467)
Reflections used for *R*_free_	308 (30)	189 (21)	163 (16)	1079 (72)	1585 (174)
*R*_work_	0.2604 (0.3063)	0.2651 (0.3778)	0.2967 (0.4073)	0.1942 (0.3242)	0.1671 (0.2556)
*R*_free_	0.3267 (0.3946)	0.2797 (0.3668)	0.3131 (0.4339)	0.2195 (0.3360)	0.1963 (0.2884)
CC (work)	0.922 (0.845)	0.955 (0.604)	0.907 (0.381)	0.960 (0.690)	0.963 (0.797)
CC (free)	0.869 (0.504)	0.906 (0.710)	0.780 (0.384)	0.958 (0.783)	0.962 (0.786)
No. of non-H atoms					
Total	2638	1657	1748	1912	2013
Macromolecules	2611	1655	1748	1785	1792
Ligands	15	0	0	13	100
Solvent	12	2	0	117	160
Protein residues	373	233	228	238	234
R.m.s.d., bonds (Å)	0.003	0.006	0.004	0.012	0.011
R.m.s.d., angles (°)	0.5	0.78	0.67	0.98	1.05
Ramachandran favoured (%)	96.46	93.07	96.90	98.31	99.14
Ramachandran allowed (%)	3.54	6.93	3.10	1.69	0.86
Ramachandran outliers (%)	0.00	0.00	0.00	0.00	0.00
Rotamer outliers (%)	0.00	0.00	0.57	0.00	0.00
Clashscore	8.41	12.16	8.19	1.75	3.87
Average *B* factor (Å^2^)					
Overall	68.78	211.57	156.62	36.76	22.36
Macromolecule	68.71	211.79	156.62	39.39	21.07
Ligands	100.52	0	0	60.41	33.39
Solvent	45.11	30.00	0	43.53	32.57
No. of TLS groups	3	ND	ND	ND	6

**Table 3:** Crystallization time, propensity and diffraction quality of 1TEL–DARPin constructs in sparse crystallization conditions (before the optimization of crystallization conditions)

Construct	Days to crystal appearance	Crystal habitus	Minor axis of largest crystal (µm)	Percentage of sparse conditions with crystals	No. of crystals diffracting X-rays	Diffraction limit† (Å)
10×His-1TEL-short-rigid-DARPin	1–2	Rod	24–72	6.25	12	6.61 (3.54–8.20)
10×His-1TEL-medium-rigid-DARPin	0.5–20	Hexagonal prism	5–64	22.2	28	6.84 (3.34–10.2)
10×His-1TEL-long-rigid-DARPin	21–30	Rod	10	2.4	0	No diffraction
10×His-1TEL-PA-DARPin	23–30	Blob	32–47	1.7	0	No diffraction
10×His-1TEL-PAA-DARPin	4–9	Blob	36–48	0.7	1	4.28
10×His-1TEL-GG-DARPin	21–30	Needle	8–40	0.7	0	No diffraction
10×His-1TEL-GGG-DARPin	7–14	Needle	15–24	2.1	1	12.8
1TEL-PA-DARPin	0.5–169	Rod	4–89	4.9	3	5.00 (3.51–8.10)
1TEL-PAA-DARPin	1–2	Needle	22–43	5.2	3	6.90 (5.40–8.20)
1TEL-GG-DARPin	17–175	Short barrels	29–41	8.0	0	No diffraction
1TEL-GGG-DARPin	5–179	Needle	8–16	5.9	ND	Not harvestable

† As not all crystals of a given construct are reasonably expected to be of the same quality, diffraction resolutions are reported as the mean, with the range given in parentheses.

**Table 4:** Diffraction and data-collection statistics of optimized crystals

Construct	No. of datasets collected	Diffraction limit of collected datasets† (Å)	Fraction of non-ice reflections indexed† (%)	Average estimated mosaicity† (°)	Final ISa of data processing†	Unit-cell solvent content† (%)
10×His-1TEL-short-rigid-DARPin (‘3-fold’; PDB entry 9znb)	3	1.85 (1.77–1.92)	84.9 (82.8–89.0)	0.40 (0.35–0.45)	28 (25–32)	45 (42–46)
10×His-1TEL-short-rigid-DARPin (‘2-fold’; PDB entry 9dvg)	2	3.53 (3.51–3.54)	63.5 (53.5–73.5)	0.35 (0.34–0.35)	23 (23–24)	56 (53–59)
10×His-1TEL-medium-rigid-DARPin (PDB entry 9dp8)	6	3.28 (2.87–4.61)	15.0 (13.0–42.5)	0.70 (0.40–1.60)	22 (10–40)	59 (56–60)
SUMO-1TEL-PA-DARPin (PDB entry 9db5)	9	2.69 (1.57–4.00)	51.2 (4.3–93.9)	0.39 (0.16–0.70)	22 (11–36)	54 (50–61)
SUMO-1TEL-GG-UBA (PDB entry 7tdy; Nawarathnage *et al.*, 2023 ▸)	9	2.01 (1.42–4.00)	31.6 (21.6–44.3)	0.55 (0.25–1.20)	23 (16–29)	44 (41–45)

† As not all crystals of a given construct are reasonably expected to be of the same quality, values are reported as the mean, with the range given in parentheses.

**Table 5:** Comparison of unique crystal contacts and total contact area per asymmetric unit across 1TEL–DARPin constructs

Construct	No. of unique crystal contacts per asymmetric unit involving the DARPin	Total area of crystal contacts involving the DARPin (Å^2^)	Solvent content (%)	Resolution (Å)
10×His-1TEL-medium-rigid-DARPin	2	199	59	3.47
10×His-1TEL-short-rigid-DARPin (‘2-fold’)	3	307	53	3.54
10×His-1TEL-short-rigid-DARPin (‘3-fold’)	3	1085	42	1.77
1TEL-PA-DARPin	4	1855	50	1.57

**Table 6:** Crystallization time, propensity and diffraction quality of 1TEL–UBA constructs NA, not applicable; ND, not determined.

Construct	Days to first crystal appearance	Crystal habitus	Minor axis of largest crystal (µm)	Percentage of sparse conditions giving crystals	No. of crystals diffracting beyond 4 Å	Diffraction limit† (Å)
1TEL-GG-UBA (PDB entry 7tdy; Nawarathnage *et al.*, 2023 ▸)	1	Hexagonal prism, tapered at one or both ends	80	3.5	10	2.2 (1.4–3.2)
1TEL-PA-UBA	1	Hexagonal prism, tapered at one or both ends	30	10	0	No diffraction
10×His-1TEL-short-rigid-UBA	1	Micro-needles	12	3.8	0	Not mountable
1TEL-long-rigid-UBA	No crystals	NA	NA	0	0	ND

† As not all crystals of a given construct are reasonably expected to be of the same quality, diffraction resolutions are reported as the mean, with the range given in parentheses.
