# Optimal 1TEL–target protein linker character is target protein-dependent. Corrigendum

**DOI:** 10.1107/S2059798326007102

**Published:** 2026-07-11

**Authors:** Maria J. Pedroza Romo, Alihikaua Keliiliki, Jacob C. Averett, Joseph F. Gonzalez, Ethan Noakes, Elijah W. Wilson, Conrad Smith, Blake Averett, Dalton Hansen, Riley Nickles, Miles Bradford, Sara Soleimani, Tobin Smith, Supeshala Nawarathnage, Prasadika Samarawickrama, Ariel Kelsch, Derick Bunn, Cameron Stewart, Wisdom Abiodun, Evan Tsubaki, Seth Brown, Tzanko I. Doukov, James D. Moody

**Affiliations:** a Department of Chemistry and Biochemistry Brigham Young University 701 East University Parkway Provo UT84602 USA; b Macromolecular Crystallography Group, Structural Molecular Biology Resource, Stanford Synchrotron Radiation Lightsource, Menlo Park, CA94025, USA

**Keywords:** TELSAM, DARPins, ETV6, designed ankyrin-repeat proteins, protein binding, linkers, 1TEL

## Abstract

The name of one of the authors in the article by Pedroza Romo *et al.*[(2026), *Acta Cryst.* D**82**, 516–532] is corrected.

## Abstract 2

The article by Pedroza Romo *et al.*[(2026), *Acta Cryst.* D**82**, 516–532] is corrected.

In the article by Pedroza Romo *et al.* (2026 ▸) the name of one of the authors was given incorrectly. The correct name is Prasadika Samarawickrama as given here.
